# Oncolytic virus encoding decorin and the CD40 ligand boosts T-cell response and achieves a durable antitumor response during breast cancer treatment

**DOI:** 10.1186/s43556-026-00570-w

**Published:** 2026-10-05

**Authors:** Yingjun Ning, Yejing Rong, Haifeng Meng, Yudong Lin, Wanqian Chen, Qiaoyun Shi, Sijia Zhang, Hongshan Li, Yuefeng Yang

**Affiliations:** 1 Centre for Medical Research, Ningbo No.2 Hospital, Ningbo, 315010 China; 2 The First Affiliated Hospital of Ningbo University, Ningbo, 315010 China; 3 Beijing Jingda Biotechnology Co. Ltd, Beijing, 102629 China

**Keywords:** Triple-negative breast cancer, Oncolytic adenoviruses, Decorin, CD40L, Combination therapy, Tumor microenvironment

## Abstract

Triple-negative breast cancer (TNBC) remains an aggressive disease with limited treatment options. Oncolytic adenoviruses represent a promising platform, but their efficacy as single agents is often insufficient. This study examined whether an oncolytic adenovirus co-delivering decorin (DCN) and CD40 ligand (CD40L), designated rAd.DCN.CD40L, could enhance antitumor activity and immune activation against TNBC. Recombinant adenoviruses expressing DCN (rAd.DCN), CD40L (rAd.CD40L), or both proteins were constructed and evaluated in breast cancer cells using cell viability, apoptosis, migration, invasion, and damage-associated molecular pattern (DAMP) release assays. Compared with rAd.DCN or rAd.CD40L alone, rAd.DCN.CD40L triggered greater apoptosis and tumor cell death and elicited more pronounced DAMP release. Co-culture experiments also revealed enhanced dendritic cell function following rAd.DCN.CD40L treatment. For in vivo evaluation, syngeneic 4T1 and EMT-6 subcutaneous tumor models and a 4T1 lung metastasis model were employed. rAd.DCN.CD40L substantially delayed tumor growth and improved survival in tumor-bearing mice. Flow cytometric and histological analyses further confirmed enhanced local and systemic T-cell responses, elevated CD8⁺ T-cell activity, and modulated inflammatory macrophage phenotypes. Mechanistically, western blot analysis showed that rAd.DCN.CD40L may contribute to extracellular matrix remodeling and the down-regulation of the expression of epithelial-mesenchymal transition (EMT)-associated genes. Notably, CD8⁺ T-cell depletion markedly attenuated the therapeutic efficacy of rAd.DCN.CD40L, demonstrating that CD8⁺ T cells are critical functional effectors of tumor control. Collectively, these findings indicate that a DCN- and CD40L-armed oncolytic adenovirus represents a promising combinatorial strategy for TNBC immunotherapy, and warrants further preclinical and clinical investigation.

**Supplementary Information:**

The online version contains supplementary material available at https://doi.org/10.1186/s43556-026-00570-w.

## Introduction

Immunotherapy has reshaped the treatment landscape for multiple cancers. However, triple-negative breast cancer (TNBC), a highly aggressive subtype, is frequently characterized by a highly immunosuppressive tumor microenvironment, which limits the generation of effective antitumor immune responses [1]. Cancer immunotherapy is a well-established treatment strategy that uses the patient’s own immune system, including both innate and adaptive immunity, to identify and eliminate cancer cells [2]. Therefore, remodeling the tumor microenvironment and establishing an effective and durable anticancer response have become major priorities in TNBC treatment [3].

Oncolytic adenoviruses represent a versatile platform for cancer immunotherapy. These viruses replicate preferentially in tumor cells, inducing direct oncolysis while largely sparing normal tissues [4–6]. Local viral infection can also release tumor-associated antigens and danger signals, which may recruit tumor-specific CD8^+^ T cells and, under favorable conditions, trigger systemic antitumor immunity [7, 8]. The induction of such T-cell responses is considered an important prerequisite for achieving abscopal antitumor activity and long-term therapeutic effects, a concept supported by clinical studies using the oncolytic herpes virus T-Vec [9, 10]. To amplify these responses, oncolytic viruses are increasingly engineered to carry immunostimulatory payloads, such as interleukin-2 (IL-2), tumor necrosis factor-α (TNF-α), and interleukin-1 (IL-1) [11–13].

Decorin (DCN) is underexpressed in many cancers and reportedly functions as a receptor tyrosine kinase inhibitor and a natural antagonist of transforming growth factor-β (TGF-β), a cytokine implicated in metastasis and immunosuppression [14, 15]. DCN also regulates tumor cell proliferation, migration, and angiogenesis, and modulates the antitumor microenvironment through inflammatory responses involving Th1 and Th2 cells [16–18]. We previously reported that DCN-expressing oncolytic viruses (rAd.DCN) exhibited antitumor responses, which were modestly enhanced by the addition of granulocyte-macrophage colony-stimulating factor (GM-CSF) [19]. In contrast to DCN, which directly affects tumor cells and stroma, CD40 ligand (CD40L) acts primarily on immune cells [20]. CD40L is a member of the tumor necrosis factor superfamily (TNFSF) [21], and the CD40-CD40L axis is considered a costimulatory pathway that promotes both humoral and cell-mediated immune responses [22]. This axis has been studied as a promising target for immunotherapy [23], as it activates dendritic cells (DCs), which in turn secrete costimulatory molecules and cytokines that can initiate antigen-specific T-cell responses [21, 24]. Both molecules have been proposed as potential targets for cancer therapy. The oncolytic adenovirus rAd.DCN.CD40L, which co-expresses decorin and CD40 ligand, was previously evaluated in a murine colorectal tumor model and was found to not only delay subcutaneous tumor growth but also inhibit liver metastasis through the activation of immune effector cells [25]. However, the immune mechanisms underlying this effect were not fully defined, and whether this dual-gene strategy can overcome the immunosuppressive microenvironment of TNBC remains unclear.

In this study, it was hypothesized that a DCN- and CD40L-armed oncolytic adenovirus could integrate direct tumor suppression with immune activation and thereby enhance antitumor efficacy in TNBC. rAd.DCN, rAd.CD40L, and rAd.DCN.CD40L were evaluated using both in vitro and in vivo experiments. Tumor cell viability, apoptosis, migration, invasion, and damage-associated molecular pattern release were assessed in vitro, while antitumor responses were evaluated in subcutaneous tumor and lung metastasis models in vivo. Moreover, the mechanisms underlying antitumor activity—including immune profiling, analysis of epithelial-mesenchymal transition (EMT)-related molecules, and CD8⁺ T-cell depletion experiments—were also investigated. rAd.DCN.CD40L treatment was associated with reduced tumor growth and increased tumor-infiltrating T lymphocytes (TILs). Compared with the single-expression counterparts, the dual-expression oncolytic virus exhibited enhanced antitumor activity, suggesting both local and systemic antitumor effects with sustained efficacy during the observation period. It is proposed that rAd.DCN.CD40L therapy may exert antitumor effects through several potential mechanisms, including direct tumor inhibition (extracellular matrix remodeling and EMT suppression), enhanced antigen presentation, modulated inflammatory macrophage phenotypes, and T-cell activation. With regard to immune activation, it was hypothesized that DCN expression in tumor cells modulates the tumor microenvironment to recruit immune cells, and that CD40L on tumor cells subsequently interacts with CD40 on immune cells, such as dendritic cells, to evoke tumor-specific immune responses. Thus, rAd.DCN.CD40L therapy may represent a promising strategy for enhancing antitumor activity and could expand the therapeutic potential of oncolytic adenoviruses.

## Results

### In vitro characterization and immunomodulatory effects of rAd.DCN.CD40L

**Fig. 1:**
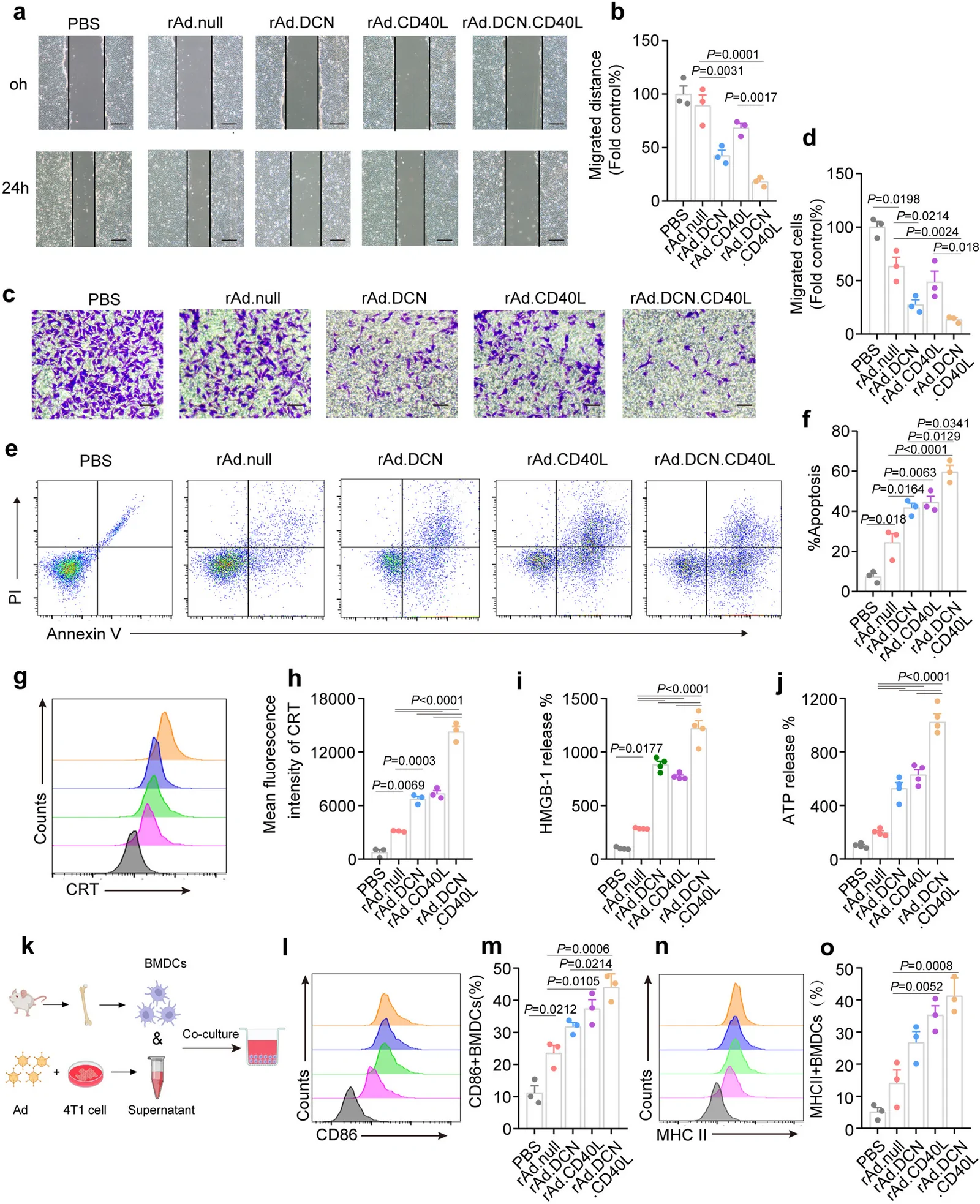
Oncolytic adenovirus rAd.DCN.CD40L suppresses tumor cell migration, invasion, and survival while promoting DAMP release and BMDC activation in vitro. **a-b** Scratching of a single layer of 4T1 cells with a sterile micropipette tip for migration observation under a microscope, scale bar = 100 μm, and statistical analysis of cell migration (*n* = 3). **c-d** Transwell assay evaluating the migratory capacity of 4T1 cells, scale bar = 50 μm, and statistical analysis of migrated cells (*n* = 3). **e–f** Representative flow cytometry plots and quantification of the percentage of tumor cell apoptosis (*n* = 3). **g-h** CRT expression in 4T1 cells from different treatment groups, determined by flow cytometry analyses (*n* = 3). **i** HMGB1 levels in the supernatant of 4T1 tumor cells after various treatments were measured by ELISA (*n* = 4). **j** Extracellular ATP levels at 48 h after the various treatments were measured by an ATP bioluminescent assay kit (*n* = 4). **k** Schematic illustration of the co-culture of BMDCs and 4T1 cell supernatant (*n* = 3). **l-o** The microenvironment and killing functions were evaluated by flow cytometry analysis of CD86 and MHC II levels in BMDCs co-cultured with the treated 4T1 cell supernatant (*n* = 3). The last column represents the combination treatment. The *P* values by one-way ANOVA with Tukey’s post hoc test. The data are shown as means ± SD of independent biological replicates. rAd, oncolytic adenovirus; PBS, phosphate-buffered saline; DCN, decorin; CD40L, CD40 ligand; DAMP, damage-associated molecular pattern; BMDCs, bone marrow-derived dendritic cells; CRT, calreticulin; HMGB1, high-mobility group protein B1; ATP, adenosine triphosphate; ELISA, enzyme-linked immunosorbent assay

rAd.DCN.CD40L is a dual-gene-expressing oncolytic adenovirus with conditional replication in tumor cells, controlled by the telomerase reverse transcriptase (*TERT*) promoter (TERTp) (Fig. S1a) [26, 27]. The oncolytic adenovirus rAd.DCN.CD40L effectively expressed both *Dcn* and *Cd40l* in breast cancer cells (Fig. S1b-c). The half-maximal inhibitory concentration (IC₅₀) determined as multiplicity of infection (MOI), was determined by cell viability assays, which revealed dose-dependent cytotoxicity across human and murine breast cancer cell lines (Fig. S1d-e). Based on these viability data, the MOI used for subsequent functional experiments was chosen to avoid significant reduction in cell viability relative to the control group. Compared with rAd.null, rAd.DCN significantly inhibited the migration of 4T1 cells, whereas rAd.DCN.CD40L exerted a stronger inhibitory effect and induced more pronounced apoptosis (Fig. 1a-f). Previous studies have indicated that both DCN and CD40L are involved in regulating tumor cell apoptosis [18, 28].

To assess changes in damage-associated molecular patterns (DAMPs) during immunogenic cell death, the translocation of calreticulin (CRT) and the extracellular release of high-mobility group protein B1 (HMGB1) and adenosine triphosphate (ATP), were detected in breast cancer cells after oncolytic adenoviral transduction. Immunogenic cell death (ICD) is known to induce antitumor immune responses within the tumor microenvironment (TME) [29]. Compared with rAd.null, rAd.DCN and rAd.CD40L significantly increased CRT translocation and HMGB1/ATP release, whereas rAd.DCN.CD40L produced the most pronounced effects (Fig. 1g-j). Together, these findings suggest that adenoviral-mediated expression of DCN or CD40L promoted DAMP release consistent with immunogenic tumor cell death.

To further evaluate ICD-induced antitumor immune activation, bone marrow-derived dendritic cells (BMDCs) were cultured with conditioned medium from oncolytic adenovirus-treated 4T1 cells (Fig. 1k). Oncolytic adenovirus treatment increased the expression of cluster of differentiation 86 (CD86) and major histocompatibility complex class II (MHC II) on DCs (Fig. 1l-o). In contrast, treatment with rAd.DCN alone did not significantly alter these maturation markers. Together, these results suggest that CD40L expression may contribute importantly to BMDC maturation, although the relative contributions of CD40L signaling, DAMP release, and viral components require further mechanistic investigation.

### Safety and systemic pharmacokinetics of oncolytic adenoviruses in the 4T1 mouse model

**Fig. 2:**
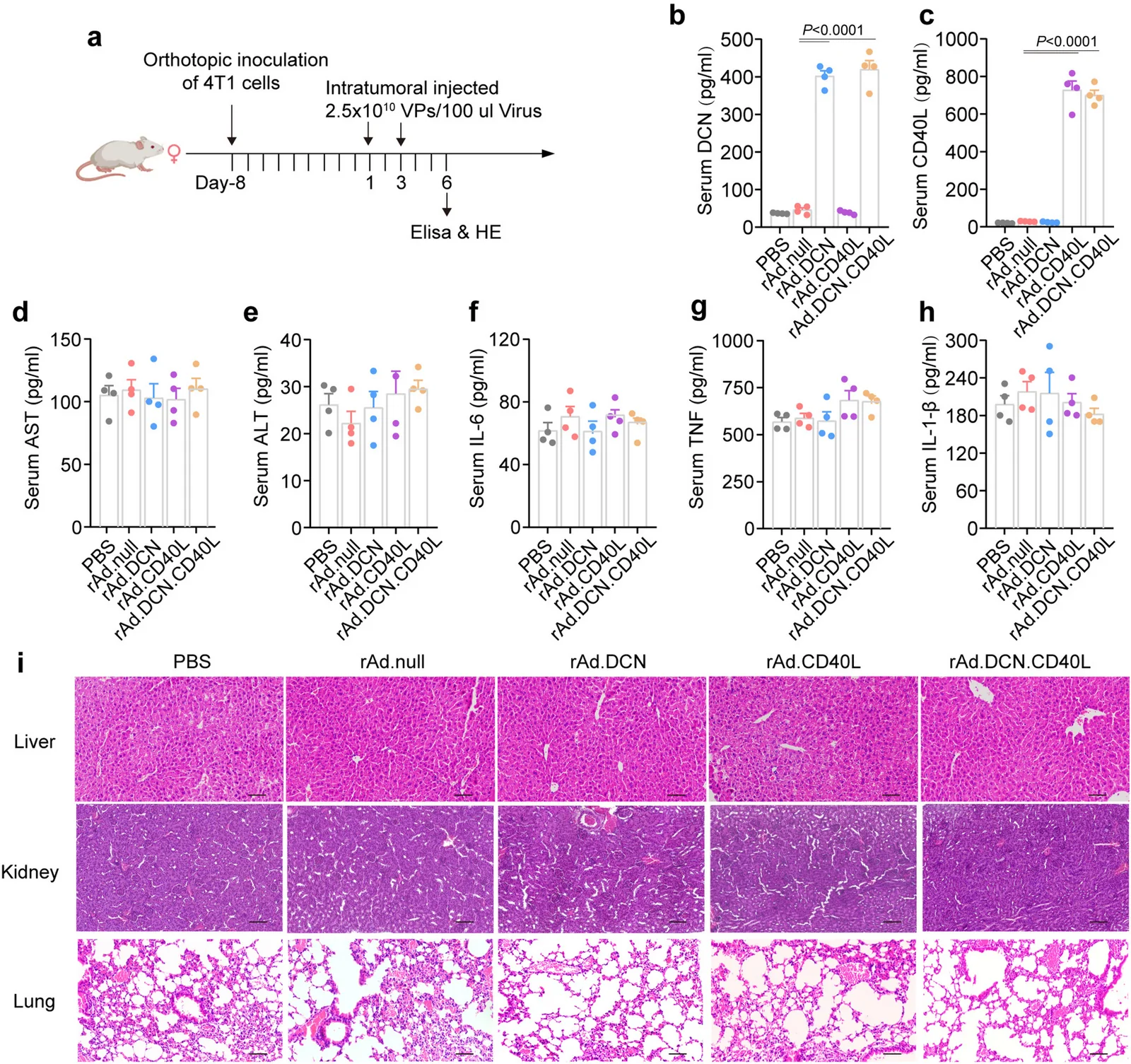
Safety and systemic pharmacokinetics analysis of the intratumoral administration of replicating adenoviruses in a 4T1 breast tumor-bearing female BALB/c mouse model.** a** Diagram illustrating the therapeutic procedures. Female BALB/c mice aged 4 − 6 weeks were implanted with 4T1 cells, followed by two treatments with different virus treatment via subcutaneous tumor injection on alternate days (*n* = 4). **b-c** Mouse blood was collected on day 3 after treatment for the measurement of DCN and CD40L production by ELISA. **d-h** Measurement of biochemical parameters, including AST and ALT levels in the blood of 4T1 tumor-bearing mice on day 3 after treatment (*n* = 4). **i** H&E staining of different tissues collected on day 3 after treatment. Scale bar = 100 μm. The last column represents the combination treatment. The *P* values were determined by one-way ANOVA with Tukey’s post hoc test. The data are shown as the means ± SD of independent biological replicates. rAd, oncolytic adenovirus; PBS, phosphate-buffered saline; DCN, decorin; CD40L, CD40 ligand; ALT, alanine aminotransferase; AST, aspartate aminotransferase; H&E, hematoxylin and eosin; TNF, tumor necrosis factor; IFN, interferon; IL, interleukin

The experimental procedure for the safety evaluation is presented in Fig. 2a. Intratumoral injection of rAd.DCN and rAd.CD40L resulted in the secretion of DCN and CD40L proteins into the serum (Fig. 2b-c). No obvious changes in aspartate aminotransferase (AST) or alanine aminotransferase (ALT) levels-markers of systemic toxicity or hepatotoxicity-were observed in the serum at 72 h after treatment with oncolytic adenoviruses (Fig. 2d-e). Similarly, no significant changes were detected in IL-6, TNF-α, or IL-1β levels after oncolytic adenoviral treatment (Fig. 2f-h). No obvious histopathological changes were detected in the liver, kidney, or lung at 3 days after treatment (Fig. 2i). The liver, kidney, and lung retained normal tissue architecture, with no obvious necrosis, edema, hemorrhage, or inflammatory infiltration. Together, these results indicate that the intratumoral delivery of oncolytic adenoviruses did not cause evident acute toxicity under the tested conditions.

### Anti-tumor efficacy of rAd.DCN.CD40L in a triple-negative breast tumor-bearing BALB/c mice model

**Fig. 3:**
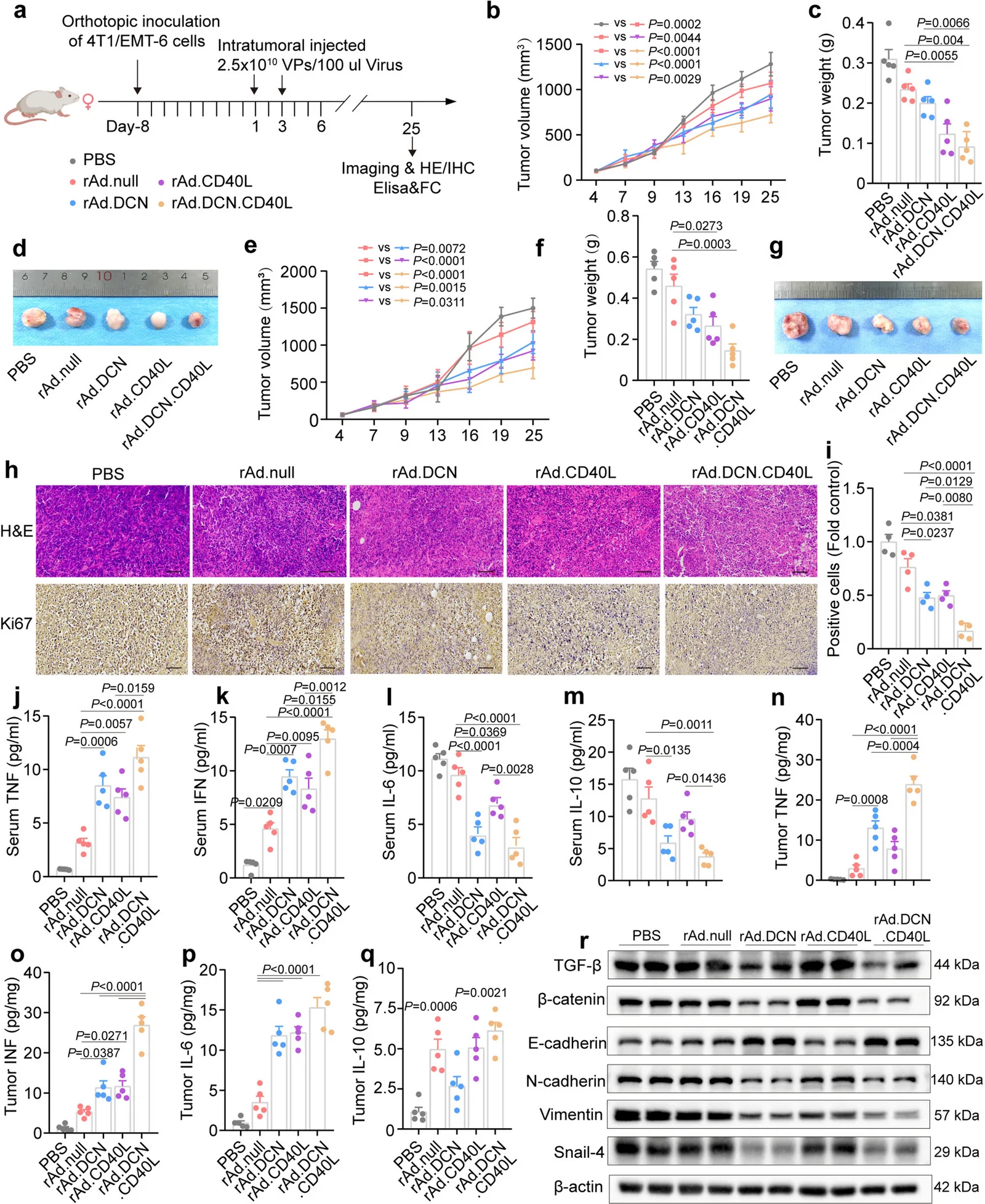
rAd.DCN.CD40L significantly inhibits tumor growth in triple-negative breast tumor-bearing female BALB/c mice. **a** Schematic diagram of the combined treatment process; **b-d** Primary 4T1 tumors were monitored by volume for 25 days, with endpoint tumor weights and representative tumor images shown (*n* = 5); **e–g** Primary EMT-6 tumors were monitored by volume for 25 days, with endpoint tumor weights and representative tumor images shown (*n* = 5); **h-i** 4T1 tumor sections stained with H&E and Ki67, statistical graph of Ki67-positive cells (*n* = 4), scale bar = 100 μm. **j-m** Quantitative analysis of serum levels of TNF-α, IFN-γ, IL-6 and IL-10 was performed on day 25 (*n* = 5). **n-q** Quantitative analysis of the intratumoral levels of TNF-α, IFN-γ, IL-6 and IL-10 was performed on day 25 (*n* = 5). **r** Western blotting of TGF-β and EMT-related molecules in tumor samples was shown (*n* = 4). The last column represents the combination treatment. The *P* values were determined by one-way ANOVA with Tukey’s post hoc test. The data are shown as the means ± SD of independent biological replicates. TGF-β, transforming growth factor-β; EMT, epithelial-mesenchymal transition

Subcutaneous 4T1 and EMT-6 breast tumor models were established, and tumor-bearing mice were randomly assigned to five treatment groups once tumors reached comparable baseline sizes (Fig. 3a). Compared with the PBS group, all oncolytic adenoviral treatments delayed tumor growth, with rAd.DCN.CD40L showing the slowest growth rate over 25 days and outperforming either single-gene treatment (Fig. 3b). Analysis of tumor weights at the endpoint further confirmed that rAd.DCN.CD40L therapy achieved the strongest tumor growth inhibition (Fig. 3c-d). Endpoint ELISA confirmed increased intratumoral expression of DCN and/or CD40L after treatment with the corresponding transgene-expressing oncolytic adenoviruses (Fig. S2a-b). Comparable antitumor efficacy was also observed in the EMT-6 TNBC model (Fig. 3e-g). Histopathological examination showed that tumors in all groups retained typical malignant morphology, including high cellularity, irregular cellular arrangement, marked nuclear pleomorphism, and atypical mitotic activity. Subsequent staining further revealed that oncolytic adenoviral treatment promoted apoptosis and inhibited tumor cell proliferation in the 4T1 model, with the most pronounced changes in the rAd.DCN.CD40L group (Fig. 3h-i).

TNF-α and IFN-γ are key mouse Th1 cytokines that contribute to antitumor immunity by enhancing cytotoxic lymphocyte responses and natural killer cell effector activity [30, 31]. By contrast, IL-6 and IL-10 are implicated in immunosuppressive regulation within the tumor microenvironment [32]. Cytokine levels in the serum and tumor tissues were analyzed by ELISA. Relative to rAd.null, rAd.DCN, rAd.CD40L, and rAd.DCN.CD40L each increased serum TNF-α and IFN-γ levels while reducing IL-6 and IL-10 levels (Fig. 3j-m). Notably, all tested cytokines were markedly upregulated by oncolytic adenoviral treatment in tumor tissues (Fig. 3n-q); however, the increase in TNF-α and IFN-γ exceeded those in IL-6 and IL-10. The strongest overall cytokine changes were observed in the rAd.DCN.CD40L group. Together, these results demonstrate that oncolytic adenoviral treatment shifted the immune response toward a more Th1-type profile. Furthermore, the DCN- and CD40L-armed oncolytic adenovirus modulated the tumor microenvironment (Fig. 3r, Fig. S3a-f). The decorin-armed oncolytic adenovirus downregulated the expression of TGF-β and β-catenin in tumors, two molecules known to be critical for tumor migration (Fig. S3a-b). In parallel, increased E-cadherin expression and reduced levels of N-cadherin, vimentin, and Snail were observed, consistent with suppression of EMT-associated changes (Fig. S3c-f).

### rAd.DCN.CD40L treatment is associated with enhanced immune-related transcriptional programs

**Fig. 4:**
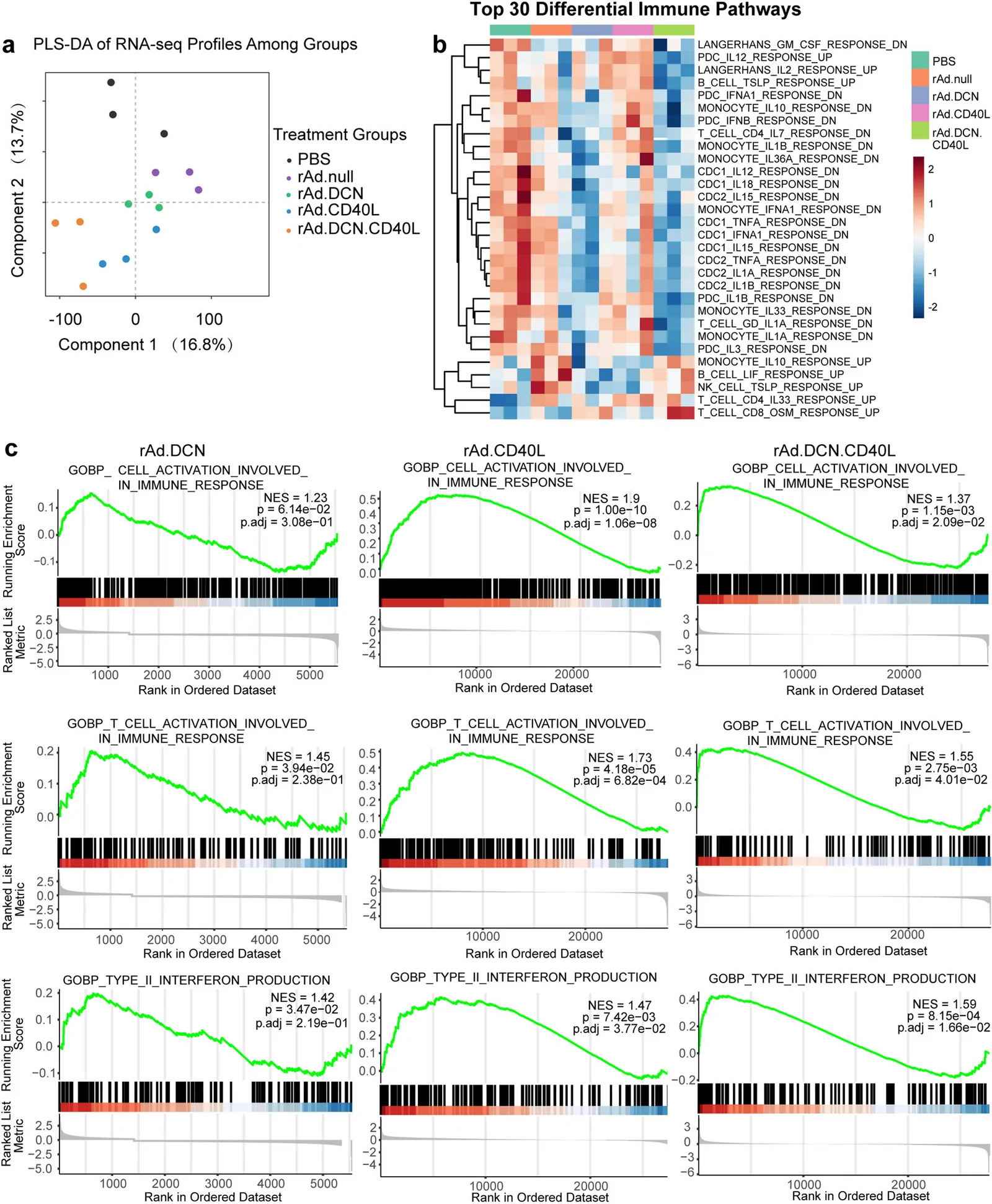
rAd.DCN.CD40L is associated with enhanced immune-related transcriptional programs. **a** PLS-DA score plot showing the separation of clusters in oncolytic viruses in different treatment groups (*n* = 3). **b** Heatmap of the results of the IPA analysis showing the signaling pathways associated with the different treatment groups (*n* = 3). **c** GSEA of the immune response in mouse 4T1 tumor tissues (*n* = 3). PLS-DA, partial least squares discriminant analysis; GSEA, Gene set enrichment analysis

Transcriptomic analysis was performed on tumor tissue, and the partial least squares discriminant analysis (PLS-DA) score plot is shown in Fig. 4a. Among all groups, the rAd.DCN.CD40L group displayed the most distinct separation from the others. The genes upregulated in the rAd.DCN.CD40L group relative to the rAd.DCN and rAd.CD40L groups were enriched in immune response-related pathways, including lymphoid, myeloid, natural killer (NK) cells, and T-cell subset signatures (Fig. 4b). Gene set enrichment analysis (GSEA) further supported the activation of immune-regulatory programs, particularly T cell co-stimulation and interferon production (Fig. 4c). Together, these transcriptomic data indicate that rAd.DCN.CD40L treatment was associated with broad activation of immune-related programs within the tumor microenvironment.

### rAd.DCN.CD40L strengthens T-cell-mediated antitumor cytotoxicity

**Fig. 5:**
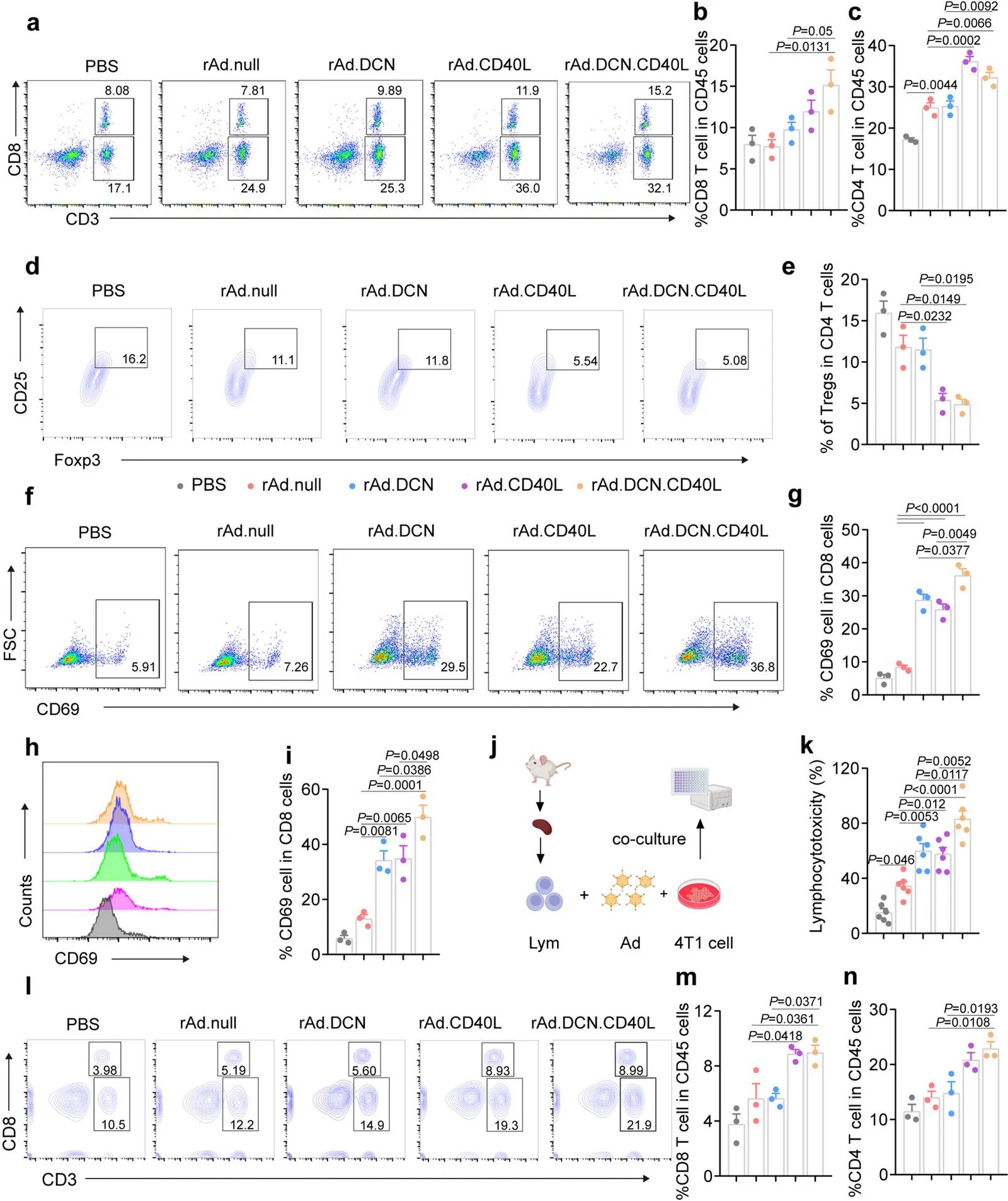
rAd.DCN.CD40L induced systemic antitumor effects in blood. Female BALB/c mice aged 4–6 weeks were implanted with 4T1 cells and treated with PBS, rAd.null, rAd.DCN, rAd.CD40L, or rAd.DCN.CD40L via subcutaneous tumor injection twice every other day. The mice were euthanized on day 14 after the first dose was administered. The number and function of immune cells in the blood and spleen were analyzed by flow cytometry. **a-c** Representative flow plots and quantification of CD4^+^ T cells and CD8^+^ T cells in the blood (*n* = 3). **d-e** Representative flow cytometry plots of Treg cells in the blood are shown (*n* = 3). **f-g** Representative flow plots and quantification of CD8^+^CD69^+^ T cells in the blood (*n* = 3). **h-i** Representative flow plots and quantification of CD8^+^CD69^+^ T cells in the spleen (*n* = 3). **j-k** Lymphocytes isolated from the spleen were co-cultured with 4T1 cells at a 100:1 ratio for 72 h to evaluate the degree of lymphocytotoxicity (*n* = 3). **l-n** Representative flow plots and quantification of CD4^+^ T cells and CD8^+^ T cells in the spleen (*n* = 3). The last column represents the combination treatment. The *P* values were determined by one-way ANOVA with Tukey’s post hoc test. The data are shown as the means ± SD of independent biological replicates. Lym, lymphocytes

To investigate the underlying immune mechanisms of the antitumor response, immune cell infiltration in the peripheral blood and spleen was analyzed on day 14 after treatment. Flow cytometry revealed that the frequency of CD8^+^ cells among CD45^+^ leukocytes was significantly increased in the peripheral blood of the rAd.DCN.CD40L-treated group (Fig. 5a-b). Similarly, CD4^+^ cell frequencies among CD45^+^ leukocytes were clearly increased in both the rAd.CD40L and rAd.DCN.CD40L groups (Fig. 5c). In addition, the frequency of circulating Tregs was reduced after treatment with rAd.CD40L and rAd.DCN.CD40L (Fig. 5d-e). CD69 expression was assessed to determine the early activation status of CD4^+^ and CD8^+^ T cell populations [33]. The percentage of CD8^+^ CD69^+^ T cells was significantly increased in the rAd.DCN, rAd.CD40L, and rAd.DCN.CD40L groups, with the greatest increase observed in the rAd.DCN.CD40L-treated group (Fig. 5f-g). A comparable increase in CD69⁺CD8⁺ T cells was observed in the spleen (Fig. 5h-i). Consistent with this, splenic T cells from treated mice exhibited enhanced antitumor activity in vitro, with the strongest effect in the rAd.DCN.CD40L group (Fig. 5j-k). Furthermore, the changes in splenic lymphocytes after viral treatment paralleled those observed within the tumor microenvironment (Fig. 5l-n). Together, these results indicate that co-expression of DCN and CD40L was associated with enhanced systemic and intratumoral T-cell responses.

### rAd.DCN.CD40L treatment promotes immune cell infiltration

**Fig. 6:**
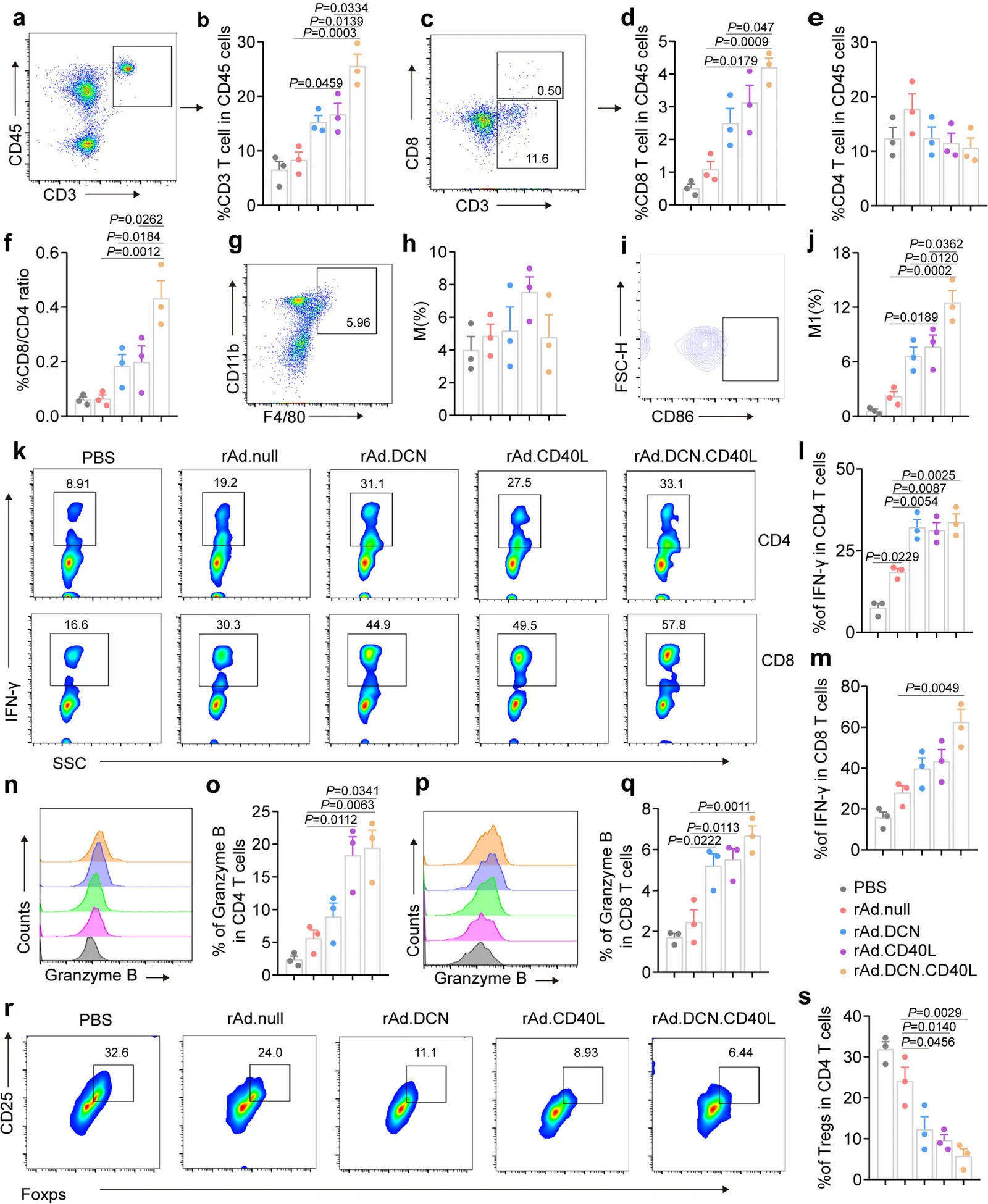
rAd.DCN.CD40L increases the number of tumor-infiltrating T lymphocytes. Female BALB/c mice aged 4–6 weeks were implanted with 4T1 cells and treated with PBS, rAd.null, rAd.DCN, rAd.CD40L, or rAd.DCN.CD40L via subcutaneous tumor injection twice every other day The mice were euthanized on day 14 after the first dose was administered. The number and function of immune cells in the TME were analyzed by flow cytometry. **a-b** Proportion of CD45^+^CD3^+^ T cells in tumors in the 4T1 tumor model (*n* = 3). **c-f** Proportion of CD8^+^ T cells and CD4^+^ T cells among CD45^+^ cells, ratios of CD8/CD4 T cells (*n* = 3). **g-j** The proportion of macrophages and M1 macrophages in tumor tissues are shown (*n* = 3). **k-m** Proportion of IFN-γ secretion by CD4^+^ T cells and CD8^+^ T cells. **n–q** Granzyme B production by CD4^+ ^T cells and CD8^+^ T cells (*n* = 3). **r-s** Flow cytometry measurement of Treg cell numbers in tumor tissue (*n* = 3). The last column represents the combination treatment. The *P* values were determined by one-way ANOVA with Tukey’s post hoc test. The data are shown as the means ± SD of independent biological replicates. TME: tumor microenvironment; M, macrophage; M1, classically activated macrophage

To characterize intratumoral T-cell responses after oncolytic adenovirus treatment, tumor-infiltrating lymphocytes were analyzed by flow cytometry. An increase in total lymphocyte infiltration (CD45^+^ CD3^+^ cells) was observed following intratumoral delivery of rAd.DCN.CD40L (Fig. 6a-b). Moreover, increased infiltration of CD8⁺ T cells was detected after treatment with rAd.CD40L and rAd.DCN.CD40L, whereas no comparable increase was observed in CD4⁺ T cells (Fig. 6c-e). Accordingly, the CD8/CD4 ratio was significantly elevated in the rAd.DCN.CD40L group (Fig. 6f). The total number of macrophages did not change significantly after oncolytic adenoviral treatment (Fig. 6g-h). However, the inflammatory macrophage subset showed an upward trend in the rAd.CD40L group, and a more pronounced increase was observed in the rAd.DCN.CD40L group (Fig. 6i-j).

Cytotoxic T cells are key effector cells in antitumor immunity, capable of directly mediating cytolytic activity against tumor cells [34]. CD8^+^ and CD4^+^ T cells are the main sources of interferon-γ (IFN-γ), which can enhance the cytotoxic function of CD8^+^ T cells and promote recognition and elimination of tumor cells, whereas granzyme and perforin can directly kill cancer cells [29, 35]. Intratumoral IFN-γ^+^ and granzyme B^+^ cells were further evaluated. Relative to rAd.null, treatment with rAd.DCN, rAd.CD40L, and rAd.DCN.CD40L significantly enhanced IFN-γ expression in CD4^+^ T cells, whereas only rAd.DCN.CD40L enhanced IFN-γ expression in CD8^+^ T cells (Fig. 6k-m). Moreover, the numbers of granzyme B CD8^+^ and CD4^+^ T cells were also increased after treatment with rAd.DCN, rAd.CD40L and rAd.DCN.CD40L, with rAd.DCN.CD40L inducing the greatest number in tumors (Fig. 6n-q). Within the tumor microenvironment, regulatory T cells (Tregs) contribute to tumor immune evasion by suppressing antitumor immune responses, thereby promoting tumor growth and metastatic progression [36, 37]. Compared with the rAd.null group, rAd.DCN, rAd.CD40L, and rAd.DCN.CD40L significantly reduced Treg infiltration (Fig. 6r-s). Together, these results indicate that rAd.DCN.CD40L promoted effector T-cell responses within tumors while decreasing the infiltration of immunosuppressive Tregs.

### CD8⁺ T cells are indispensable for rAd.DCN.CD40L-mediated reverse of T cell exhaustion and suppression of tumor growth

**Fig. 7:**
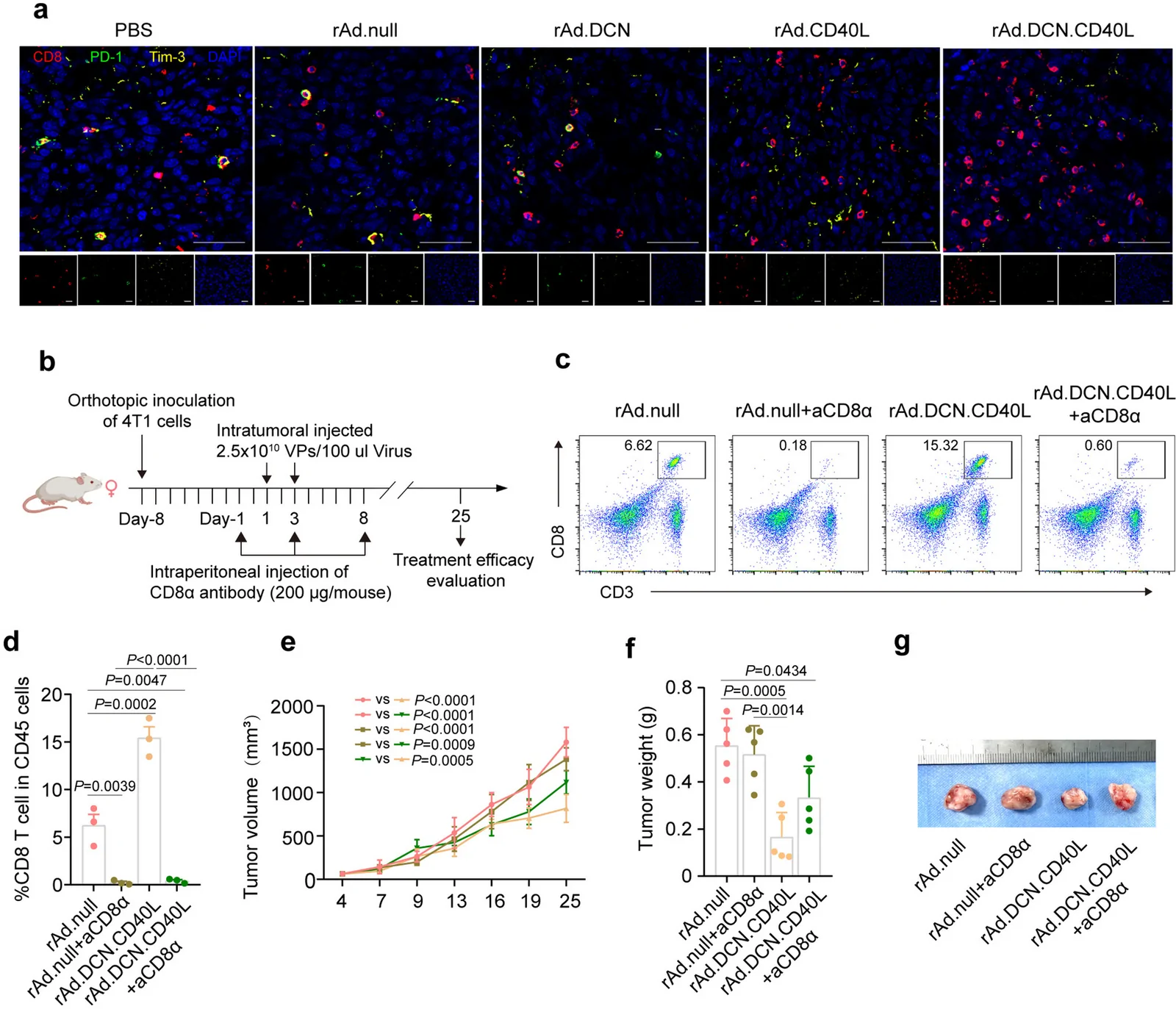
rAd.DCN.CD40L relieves T cell exhaustion to exert anti-tumor effects dependent on CD8⁺ T cells. **a** Representative images of frozen tumor sections stained for CD8 (red), PD-1 (green), TIM-3 (yellow), and DAPI (blue). Scale bar = 50 μm. **b** Schematic diagram of the progression of CD8⁺ T cell exhaustion in the 4T1 mouse models. **c-d** Flow cytometry validation of CD8⁺ T cell depletion efficiency in the peripheral blood (*n* = 3). **e–g** Tumor volume and weight at the endpoint (day 25) (*n* = 5). The last column represents the combination treatment. The *P* values were determined by one-way ANOVA with Tukey’s post hoc test. The data are shown as the means ± SD of independent biological replicates. PD-1, programmed cell death protein 1; TIM-3, T cell immunoglobulin and mucin domain-containing protein 3; aCD8α, anti-mouse CD8α

To evaluate the functional phenotype of tumor-infiltrating CD8⁺ T cells, immunofluorescence staining for CD8, programmed cell death protein 1 (PD-1), and T-cell immunoglobulin and mucin domain-containing protein 3 (TIM-3) was performed on frozen tumor sections. Compared with the control groups, rAd.DCN.CD40L treatment increased CD8⁺ T cell accumulation within tumors (Fig. 7a). Notably, PD-1 and TIM-3 expression was reduced relative to the single-gene vector groups, indicating a less exhausted CD8⁺ T-cell phenotype. To assess the functional involvement of CD8^+^ T cells in the rAd.DCN.CD40L-mediated antitumor activity, in vivo CD8^+^ T-cell depletion assays were performed (Fig. 7b). Depletion of > 95% of CD8^+^ T cells was achieved in anti-CD8α-treated mice (Fig. 7c-d). Consistent with the above results, rAd.DCN.CD40L treatment significantly reduced tumor volume and weights compared with rAd.null treatment. However, these antitumor effects were diminished following CD8⁺ T-cell depletion (Fig. 7e-g). These findings indicate that CD8^+^ T cells play a critical role in the antitumor activity of rAd.DCN.CD40L. Overall, both DCN- and CD40L-modified oncolytic adenoviruses enhanced adaptive antitumor immunity, and the dual-gene combination exerted a more potent effect.

### Distant antitumor effect of rAd.DCN.CD40L after intratumoral injection

**Fig. 8:**
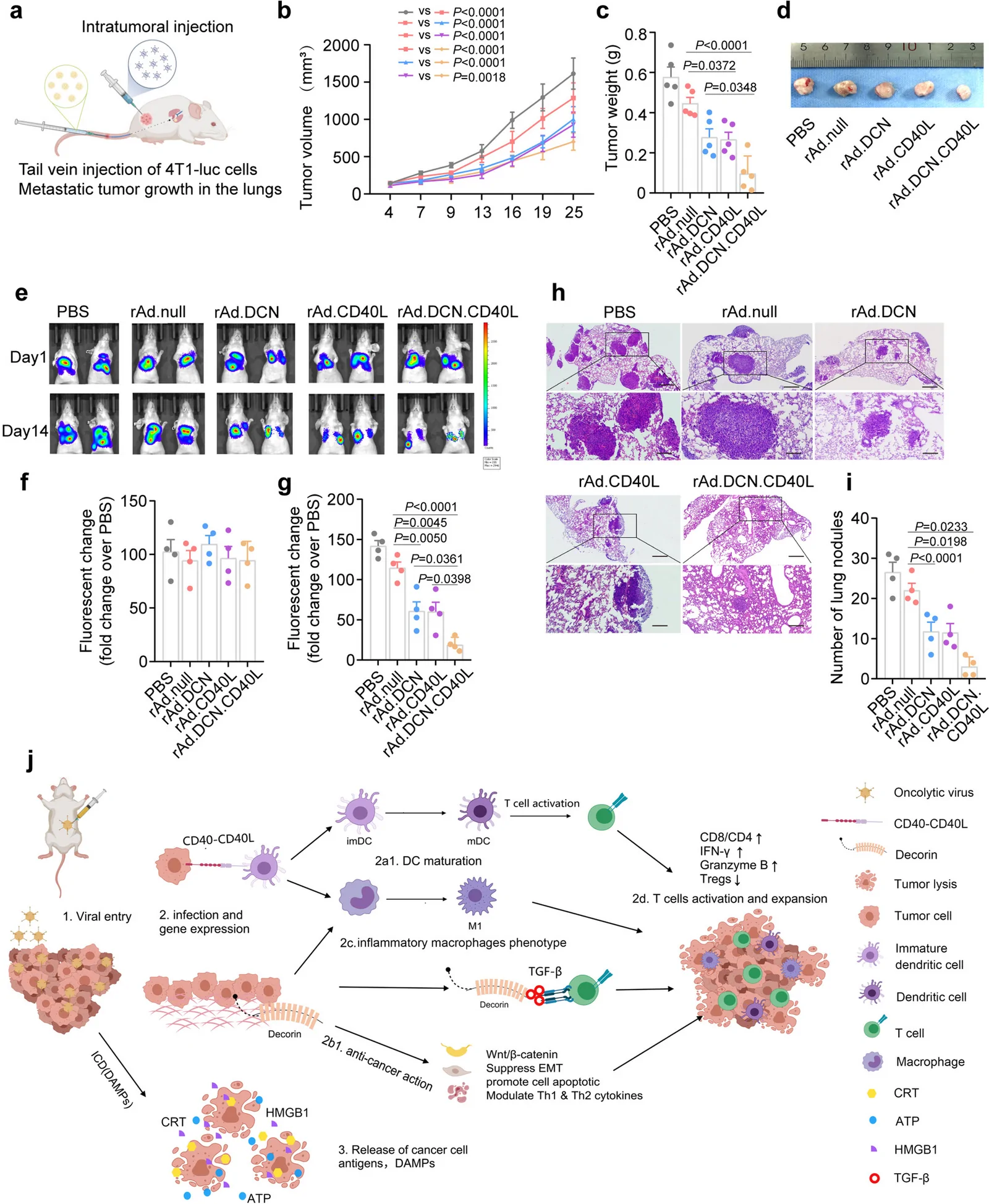
rAd.DCN.CD40L suppresses tumor metastasis and enhances systemic antitumor immunity. **a** Diagram of the 4T1-Luc lung metastasis model. When the 4T1 tumors were inoculated subcutaneously until the tumor volume reached 200 mm^3^, lung metastasis was established by tail-vein injection of 5 × 10^5^ 4T1-Luc cells (*n* = 5). **b**-**d** 4T1 tumors were monitored by volume for 25 days, with endpoint tumor weights and representative tumor images shown (*n* = 5). **e–g** Live animal bioluminescent images. The images show entrapped 4T1-Luc cells at 2 h and 14 days after tail-vein injection in the individual groups (*n* = 4). **h-i** H&E staining of lung tissue (*n* = 4); scale bars = 400 μm (top) and 100 μm (bottom) (*n* = 4). **j** Schematic diagram illustrating the mechanism of action after rAd.DCN.CD40L infection. The last column represents the combination treatment. The *P* values were determined by one-way ANOVA with Tukey’s post hoc test. The data are shown as the means ± SD of independent biological replicates

To evaluate whether rAd.DCN.CD40L was associated with systemic antitumor immunity, a mouse subcutaneous 4T1 tumor model was established and treated with intratumoral injection of oncolytic adenoviruses, followed by tail-vein injection of 4T1-Luc cells to generate pulmonary metastases (Fig. 8a). Consistent with the data described above, treatment with rAd.DCN, rAd.CD40L, and rAd.DCN.CD40L significantly inhibited subcutaneous tumor growth (Fig. 8b-d). These treatments also reduced the lung tumor burden at 14 days after treatment, as assessed by bioluminescence imaging and histopathological analysis, with the strongest effect observed in the rAd.DCN.CD40L group (Fig. 8e-i). Flow cytometry further showed an increased proportion of circulating CD3⁺CD8⁺ T cells (Fig. S4a-b) and CD3⁺CD4⁺ T cells (Fig. S4c) after rAd.DCN.CD40L treatment. Together, these findings indicate that local rAd.DCN.CD40L treatment was associated with reduced pulmonary metastasis and enhanced systemic CD8⁺ T-cell responses.

## Discussion

The present study provides evidence for the oncolytic adenovirus co-expressing decorin and CD40 ligand, rAd.DCN.CD40L, as a potential oncolytic immunotherapy candidate for TNBC. Oncolytic adenoviruses can directly lyse tumor cells while also acting as vectors for therapeutic gene delivery and immune activation [38]. Based on our previous evidence showing the favorable safety profile and retained oncolytic potency of these vectors [25], three recombinant viruses-rAd.DCN, rAd.CD40L, and rAd.DCN.CD40L-were constructed and evaluated in TNBC models. Compared with individual treatments, rAd.DCN.CD40L significantly enhanced in vitro and in vivo cytotoxicity against tumor cells. In addition, it effectively suppressed tumor growth and reduced metastasis in TNBC models, while blocking tumor-promoting signaling pathways and activating immune responses.

Dual targeting of the DCN-mediated signaling pathway and the CD40-CD40L co-stimulatory axis may help remodel the immunosuppressive microenvironment of TNBC and improve therapeutic responses in cases with limited sensitivity to existing immunotherapeutic approaches. DCN exerts its tumor-suppressive function by targeting and blocking key signaling pathways (e.g., TGF-β, Met, Wnt/β-catenin, and VEGF) that are involved in tumor immune evasion and metastasis [18, 39]. In parallel, activation of the CD40-CD40L axis enhances antitumor immunity by activating DCs and subsequently promoting T-cell responses [40]. CD40L-based combinations, such as CD40L with 4-1BBL, have shown therapeutic potential in pancreatic cancer, while DCN combined with NK cells has been shown to enhance antitumor responses in colorectal cancer models [41, 42]. However, whether DCN and CD40L can act synergistically in the immunosuppressive TNBC microenvironment remains unclear. The present study builds on previous work by demonstrating that an oncolytic adenovirus co-expressing DCN and CD40L exerts potent antitumor activity, and further clarifies the immune-activating mechanisms underlying this effect. These findings provide mechanistic insight that may guide the development of improved oncolytic virus-based immunotherapeutic strategies for TNBC.

Mechanistically, rAd.DCN.CD40L treatment may promote immunogenic cell death, as indicated by enhanced DAMP release. Relative to rAd.DCN and rAd.CD40L, rAd.DCN.CD40L induced more pronounced DAMP-associated changes, suggesting that co-expression of DCN and CD40L may further enhance this process and amplify the immunogenic effects of oncolytic viral infection. The increase in DAMP release is therefore likely attributable to the combined effects of adenovirus-mediated oncolysis, DCN/CD40L-mediated modulation, and tumor cell damage, rather than to a single mechanism. These DAMP signals can be detected by antigen-presenting cells and may promote the differentiation of naive CD8^+^ T cells into cytotoxic effector cells, which subsequently infiltrate tumors and mediate antitumor immunity [38]. Consistent with this, supernatants from rAd.DCN.CD40L-infected 4T1 cells enhanced DC activation in vitro. This experimental setup more closely mimics the actual conditions within the tumor microenvironment, as intratumoral viral injection primarily results in tumor cell infection. The resulting high transgene expression, combined with oncolytic effects and the subsequent release of viral particles, DAMPs, and tumor antigens, likely explains the enhanced activation potential of immature DCs in co-culture. However, the relative contributions of each factor remain to be determined through targeted blocking experiments in future studies. Further investigations using CD40-CD40L blockade and HMGB1/ATP pathway inhibition will be required to clarify their individual roles.

T-cell recruitment and activation are essential for effective tumor immunotherapy [43]. rAd.DCN.CD40L treatment increased T-cell infiltration and enhanced the activation status within the tumor microenvironment, as indicated by an elevated CD8/CD4 ratio, increased tumor antigen-specific CD8⁺ T cells, and increased IFN-γ levels. Similar immune activation was observed in splenocytes and peripheral blood, accompanied by a reduction in Treg frequency. In addition, enhanced infiltration of macrophages and T-cells was detected in tumor tissues. A CD8⁺ T-cell depletion experiment further confirmed that CD8⁺ T cells serve as critical effector mediators in rAd.DCN.CD40L-induced tumor suppression. Taken together, the in vitro results demonstrated significant activation of DCs following combination treatment, whereas in vivo studies revealed increased accumulation of macrophages and T cells within the tumor microenvironment. These findings suggest that rAd.DCN.CD40L-induced tumor suppression is associated with coordinated activation of the DC-macrophage-T-cell immune axis. Building upon the oncolytic foundation of the adenovirus, these integrated effects—direct inhibition of tumor-promoting signaling pathways and amplification of the immune response—collectively strengthened tumor suppression (Fig. 8j). Of note, local administration of rAd.DCN.CD40L effectively delayed the progression of distant tumors. This observation is consistent with previous evidence demonstrating that intratumoral administration of recombinant oncolytic adenoviruses can induce systemic antitumor immunity and suppress untreated lesions [44, 45]. In the present study, this systemic effect was accompanied by enhanced activation of CD8⁺ T cells in the lungs, suggesting a potential role in limiting metastatic progression. These findings are particularly relevant for TNBC, where recurrence and metastasis remain major clinical challenges. Thus, rAd.DCN.CD40L may provide a basis for developing multifunctional oncolytic virotherapies capable of inducing both local tumor control and systemic immune responses.

However, this study has several limitations. First, the experiments were conducted mainly in murine tumor models and cultured cell systems, which may not fully recapitulate the immune complexity of human breast cancer. More clinically relevant platforms, including humanized mouse models and immunocompetent large-animal models, should be used in future studies to more accurately evaluate the therapeutic potential of gene-armed oncolytic adenoviruses. Second, the sample size in several experiments was relatively limited, which may reduce statistical power and call for more cautious interpretation of borderline or non-significant findings. Future studies with larger cohorts and prospectively powered designs are needed to further confirm the robustness of these findings. Third, clinical validation was not performed in patient samples or clinical cohorts; thus, the translational feasibility of rAd.DCN.CD40L remains to be determined. Additionally, subsequent research could explore the combination of rAd.DCN.CD40L with inhibitors targeting PD-L1, T-cell immunoreceptor with Ig and ITIM domain (TIGIT), and cytotoxic T lymphocyte-associated antigen 4 (CTLA-4). Such a combination may offer a more effective therapeutic strategy for complex and refractory breast cancer, ultimately improving patient survival and quality of life.

## Conclusions

This study demonstrated that the combination of DCN and CD40L within a single oncolytic adenovirus vector elicited potent antitumor activity. Compared with rAd.DCN or rAd.CD40L alone, rAd.DCN.CD40L induced stronger immune-mediated clearance of tumor cells in both in vitro and in vivo settings. The release of DAMPs likely contributed to immune cell recruitment into the tumor microenvironment and facilitated the initiation of tumor antigen-specific T-cell responses. Both rAd.DCN and rAd.CD40L exerted tumor-inhibitory effects as individual treatments, while rAd.DCN.CD40L showed greater antitumor efficacy than either single-gene treatment group. Oncolytic adenoviruses armed with DCN and CD40L may therefore represent a promising therapeutic strategy for triple-negative breast cancer, a possibility that warrants further evaluation in well-designed clinical trials.

## Materials and methods

### Cell lines

The cell lines used in this study comprised HEK-293 human embryonic kidney cells, 4T1 mouse mammary carcinoma cells, MCF-7 cells, EMT-6 cells, and 4T1-Luc cells. All cell lines were obtained from the American Type Culture Collection (ATCC, USA). Before use, cell line identity was confirmed by short tandem repeat (STR) profiling, and all cell lines tested negative for mycoplasma contamination. HEK-293 and MCF-7 cells were maintained in Dulbecco’s Modified Eagle Medium (DMEM) containing 10% fetal bovine serum (FBS) and 1% penicillin/streptomycin. EMT-6, 4T1, and 4T1-Luc cells were maintained in high-glucose RPMI-1640 with 10% FBS and 1% penicillin/streptomycin. All media components were purchased from Gibco. Standard culture conditions were 37 ℃, 5% CO_2_, and a humidified atmosphere.

### Animal experiments

Animal experiments were approved by the Institutional Animal Care and Treatment Committee of Guoke Ningbo Life Science and Health Industry Research, and all procedures followed the approved animal care and use regulations. Female BALB/c mice (4–6 weeks old, weighing 18–20 g) were obtained from Beijing Vital River Laboratory Animal Technology Co., Ltd. (Zhejiang, China) and housed under specific pathogen-free (SPF) conditions at 22 ± 2 ℃ with 50 ± 10% relative humidity and a 12-h light/dark cycle. Mice had free access to sterilized food and water and were acclimatized for 1 week before experiments.

Syngeneic tumor models were established using 4T1 or EMT-6 cells to evaluate the antitumor effects of the oncolytic virus treatment groups. For the subcutaneous tumor model, 1 × 10⁶ 4T1 or EMT-6 cells suspended in 100 μL of PBS were inoculated subcutaneously into the right flank of each mouse. The lung metastasis model was generated by intravenous injection of 1 × 10⁶ 4T1-Luc cells via the tail-vein. Once subcutaneous tumors reached approximately 80–100 mm^3^, mice were randomly assigned (using a random number table) to different treatment groups (5 mice per group). Tumors were injected intratumorally with 50 μL of the solution containing 2.5 × 10^10^ viral particles (VPs) of the specified adenovirus or the same volume of PBS. A total of two treatments were administered at 2-day intervals. Tumor volume was monitored every 3–4 days by caliper measurement and calculated using the formula: volume = 0.5 × length × width^2^. Animals were euthanized by CO₂ asphyxiation when predefined humane endpoints were reached, including obvious clinical decline, acute body weight loss, or tumor burden greater than approximately 2000 mm^3^. Complete tumor regression was defined as the absence of palpable tumors. For safety evaluation, liver, kidney, lung, as well as blood samples were collected 2 days after after the final injection. Immune cell infiltration in tumor tissues, the peripheral blood, and the spleen was analyzed by flow cytometry.

For in vivo CD8⁺ T cell depletion, an anti-mouse CD8α blocking antibody (clone 2.43, Bio X Cell) or a control IgG isotype antibody (Bio X cell) was administered at 200 µg in 0.1 mL PBS one day before viral injection and every 7 days thereafter at the same dose. Depletion efficiency was assessed by flow cytometry of splenocytes on day 14.

### Analysis of viral replication and cytotoxicity

Cells were seeded in 6-well plates at a density of 2 × 10^5 ^cells/well and infected the next day with adenoviruses at 2.5 × 10^4^ VPs/cell. Cells were collected at 3 h and 48 h post-infection. To collect released viral particles, the entire cell cultures were transferred to fresh tubes and subjected to three freeze-thaw cycles. After this procedure, the virus-containing supernatants were harvested and used for viral genome extraction. For analysis of viral genome copy number, total DNA was prepared using a Blood Culture Cell Genomic DNA Extraction Kit (Vazyme, DC112) following the manufacturer’s instructions.

To conduct cytotoxicity assays, 1 × 10^3^ cells were seeded per well in 96-well plates and, after 24 h, infected with adenoviruses at doses ranging from 2 to 1.25 × 10^5^ VPs/cell in 100 μL of culture medium. At 7 days post-infection, cell viability was measured using the CellTiter 96® AQueous One Solution Cell Proliferation Assay (MTS). Briefly, 20 μL MTS reagent was added to each well, and the plates were incubated at 37 ℃ with 5% CO₂, followed by absorbance measurement at 490 nm using a Bio-Rad Model 680 microplate reader. Apoptotic cells were quantified by flow cytometry after Annexin V-FITC/PI staining. Detailed procedures for the apoptosis, migration, and invasion assays are provided in the supplementary methods.

### Histological and immunostaining analyses

Tumor samples were fixed in 4% paraformaldehyde and processed for either paraffin or OCT embedding. For hematoxylin and eosin (H&E) and immunohistochemistry (IHC) analyses, paraffin-embedded tissues were sectioned at 4 μm thickness, followed by routine deparaffinization and rehydration. During IHC staining, heat-induced antigen retrieval was performed, followed by blocking with 5% bovine serum albumin (BSA) for 1 h and overnight incubation at 4 ℃ with the indicated primary antibody, including anti-Ki-67 (28074–1-AP; Proteintech). Signals were developed using an horseradish peroxidase (HRP)/3,3′-diaminobenzidine (DAB) detection kit (ab64264; Abcam).

For immunofluorescence, OCT-embedded tissues were sectioned at 8 μm and stained using the Absin four-color multiplex immunofluorescence (mIHC) kit (abs50012, Absin). The following primary antibodies were used: anti-CD8α (29,896–1-AP, Proteintech), anti-PD-1 (66,220–1, Proteintech), anti-TIM-3 (11,872–1-AP, Proteintech). Each primary antibody was applied sequentially, followed by incubation with HRP-conjugated secondary antibodies and tyramide signal amplification (TSA). Nuclei were counterstained with DAPI, and multispectral images were captured using a Leica STELLARIS STED confocal microscope. Images were analyzed using LAS X software (Leica Microsystems).

### Detection of damage-associated molecular patterns (DAMPs)

The release of the DAMPs calreticulin (CRT), adenosine triphosphate (ATP), and high-mobility group box 1 (HMGB1) was assessed after adenoviral treatment. For CRT detection, cells were plated in 6-well plates at a density of 2.5 × 10^5^ cells/well and treated with adenoviruses at 1000 VPs/cell for 48 h. After treatment, cells were collected and washed twice with PBS. The samples were incubated with Alexa Fluor 647-conjugated anti-CRT antibody (ab196159, Abcam) for 40 min at 4 ℃. CRT surface exposure was quantified by flow cytometry and expressed as the proportion of CRT-positive cells. For ATP and HMGB1 assays, cells were seeded into 96-well plates at 5 × 10^3^ cells/well and treated with adenoviruses at 1000 VPs/cell. After 48 h, culture supernatants were collected. Extracellular ATP was measured using an ATP bioluminescence assay kit (S0026, Beyotime), and HMGB1 levels were quantified using a commercial ELISA kit following the manufacturers’ protocols.

### Bone marrow-derived dendritic cells (BMDCs) generation and maturation assay

BMDCs were isolated from the femurs and tibiae by PBS flushing and gently dispersed with an 18G needle. After centrifugation at 400 × g for 5 min, erythrocytes were lysed with ammonium-chloride-potassium (ACK) lysis buffer. The remaining cell suspension was passed through a 70-μm nylon mesh to remove debris and aggregates, followed by centrifugation at 600 × g for 5 min at 4 ℃. For BMDC generation, cells were resuspended at 1 × 10⁶ cells/mL in complete medium supplemented with 10 ng/mL granulocyte–macrophage colony-stimulating factor (GM-CSF) and 5 ng/mL interleukin-4 (IL-4). The cells were plated in 10-cm dishes with 10 mL medium per dish and maintained at 37 ℃ in 5% CO₂ for 3 days, followed by medium replacement and a further 3-day culture. To evaluate DC maturation and activation, BMDCs were collected and co-cultured with cell culture supernatants from adenovirus-treated cells or with control supernatants for 48 h. Cells were then collected and stained with PerCP anti-mouse CD86 (105,025, BioLegend) and FITC anti-MHC II (107,605, BioLegend) antibodies. Flow cytometry was used for analysis, and the results were expressed as ΔMFI.

### Flow cytometry

Tissue samples were cut into small pieces and enzymatically dissociated in DMEM supplemented with 200 μg/mL DNase I and 1 mg/mL type I collagenase at 37 ℃ for 30 min. The digested tissue was gently pipetted to generate a single-cell suspension, which was subsequently passed through a 70-μm cell strainer. For surface staining analysis, cells were labeled with fluorophore-conjugated antibodies for 30 min, washed twice, and resuspended in PBS before flow cytometric acquisition. For intracellular staining, surface-labeled cells were fixed and permeabilized using a BD fixation/permeabilization kit (562,574), followed by incubation with intracellular antibodies according to the supplied protocol.

The following antibodies used for flow cytometry were obtained from BioLegend and are listed as follows: anti-mouse CD45 (FITC, 157,214), anti-mouse CD3 (APC, 100,236), anti-mouse CD4 (APC/Fire 750, 100,460), anti-mouse CD8 (PerCP, 100,731), anti-mouse IFN-γ (PE, 505,807), anti-mouse Granzyme B (APC, 372,203), anti-mouse Foxp3 (PE, 118,903), anti-mouse CD86 (PerCP, 105,025), anti-mouse CD11c (PE, 117,307), anti-mouse F4/80 (Alexa Fluor 647, 123,121). Anti-mouse CD25 (APC,17–0257-41) was purchased from BD Biosciences.

### Molecular expression analyses

Real-time quantitative PCR (RT‒qPCR) and western blot analysis were conducted as described in our previous study with minor modifications [46]. Total RNA was isolated with the RNeasy Mini Kit (Qiagen, Hilden, Germany), and cDNA was then prepared using ReverTra Ace® qPCR RT Master Mix with gDNA Remover (TOYOBO Life Science) following the manufacturer’s protocol. The primers used for target gene amplification are listed below: *DCN* F: TCTCCGCAGTTGGGCAAAATGAC, R: TGGCAGAACGCACATAGACACATC;

*CD40L* F: GAAATGCAAAGAGGTGATGAGG, R: TCAGCTGTTTCCCATTTTCAAG; *GAPDH* F: AGGTCGGTGTGAACGGATTTG, R: GGGGTCGTTGATGGCAACA. GAPDH served as the internal control, and relative mRNA levels were calculated using the comparative Ct (2^−ΔΔCt^) method. RT-qPCR was performed on an Applied Biosystems StepOne Real-Time PCR System (Foster City, CA, USA) with SYBR Premix Ex Taq II (Takara Bio). For protein analysis, total protein was isolated with RIPA buffer supplemented with protease and phosphatase inhibitors, separated by SDS–PAGE, and transferred onto PVDF membranes (Pall Corporation, Port Washington, NY, USA). After blocking with 5% BSA, membranes were incubated with the indicated primary and secondary antibodies. Protein bands were visualized and quantified using the LI-COR Odyssey infrared imaging system (LI-COR biosciences, Lincoln, NE, USA). β-actin served as the loading control. The following antibodies were used: EMT Antibody Sampler Kit (Cell Signaling Technology, 9782) and TGF-β antibody (Proteintech, 81,746–2-RR) were used.

### RNA-seq data processing and bioinformatic analysis

RNA-seq was conducted with three independent biological replicates in each group (*n* = 3). Gene expression was quantified as transcripts per million (TPM), and entries sharing the same gene symbol were averaged. Global transcriptomic differences among groups were visualized by partial least squares discriminant analysis (PLS-DA). Pathway activity was quantified by single-sample gene set enrichment analysis (ssGSEA) using the GSVA package in R (method = "ssgsea"; alpha = 0.25, normalize = TRUE) with mouse gene sets from MSigDB v2025.1. Group differences in pathway scores were assessed by one-way ANOVA followed by Benjamini–Hochberg (BH) correction. Pathways were ranked by eta-squared (η^2^) effect size, and the top 30 were visualized in a row-standardized heatmap. Pairwise gene-level comparisons were performed using limma package with empirical Bayes moderation. Genes were ranked by model-derived fold change for preranked GSEA using clusterProfiler with the fgsea algorithm and the MSigDB Gene Ontology Biological Process collection (m5.go.bp.v2025.1.Mm.symbols.gmt). Statistical significance was defined as BH-adjusted *P* < 0.05. For representative enriched pathways, normalized enrichment scores (NES), nominal *P* values, and adjusted *P* values were reported.

### Statistical analysis

Quantitative results are shown as mean ± standard deviation (SD) based on at least three independent experiments. Comparisons between two groups were performed using a two-tailed unpaired Student’s t-test, whereas datasets containing multiple groups were analyzed by one-way ANOVA followed by Tukey’s post hoc test. Tumor growth curves were analyzed using two-way repeated-measures ANOVA to assess treatment effects, time, and their interaction. Statistical analyses were performed with GraphPad Prism 9, and *P* < 0.05 was regarded as statistically significant. Sample sizes were determined based on previous studies using comparable tumor models, preliminary experiments, experimental feasibility, and animal welfare considerations. No formal a priori power analysis was performed. Details regarding biological replicate numbers and animal sample sizes are stated in the respective figure legends.

## Supplementary Information

Supplementary Material 1.

## Data Availability

The Bulk RNA-seq data generated in this study have been deposited in NCBI under accession number [PRJNA1515505]. Other data supporting the findings of this study are included in the article and Supplementary Information.
