# Supplementary material for: Oncolytic virus encoding decorin and the CD40 ligand boosts T-cell response and achieves a durable antitumor response during breast cancer treatment
Source: Mol Biomed. 2026 Oct 5;7:195. doi: 10.1186/s43556-026-00570-w (PMC13635066; doi:10.1186/s43556-026-00570-w)
Supplement: Supplementary file 1 [file 43556_2026_570_MOESM1_ESM.doc]

**Supporting Information**

Oncolytic virus encoding decorin and the CD40 ligand boosts T-cell response and achieves a durable antitumor response during breast cancer treatment

Yingjun Ning^1†^, Yejing Rong^1†^, Haifeng Meng^2^, Yudong Lin^1^, Wanqian Chen^1^, Qiaoyun Shi^1^, Sijia Zhang^1^, Hongshan Li^1*^, Yuefeng Yang^1,3*^.

^1^ Centre for Medical Research, Ningbo No.2 Hospital, Ningbo 315010, China

^2^ The First Affiliated Hospital of Ningbo University, Ningbo 315010, China.

^3^ Beijing Jingda Biotechnology Co. Ltd, Beijing 102629, China.

† Yingjun Ning and Yejing Rong contributed equally to this work.

*Correspondence:

Hong-Shan Li

lihongshan_1982@126.com

Yue-Feng Yang

yuefengyang1981@163.com

Full list of author information is available at the end of the article

**Supplementary methods**

**Apoptosis assay**

Detection of apoptosis was conducted using a FITC Annexin V Apoptosis Detection Kit I (BD Pharmingen, 556547, San Diego, CA, USA) following the manufacturer's protocol. Cells were seeded in 6-well plates at 2.5 × 10^5^ and treated with adenoviruses at 1000 VPs/cell for 48 h. Thereafter, 1 × 10⁵ cells were harvested and resuspended in 500 μL binding buffer. Then, 5 μL of Annexin V-FITC and 10 μL of PI were added, and the samples were incubated for 15 min at room temperature in the dark. Apoptotic cells were quantified within 1 h by flow cytometry; data were acquired on a flow cytometer (Beckman Coulter) using CytExpert software and analyzed with FlowJo.

**Transwell assay**

Transwell migration assays were carried out using 8-μm pore inserts (Corning, NY, USA). Adenovirus-treated cells were seeded into the upper chamber at 2 × 10⁴ cells/well in serum-free medium, while medium supplemented with 10% FBS was placed in the lower chamber. After 24 h, cells remaining on the upper surface were removed. Migrated cells on the lower membrane surface were fixed and stained with 0.1% crystal violet (Beyotime, C0121). Eight randomly selected fields were photographed and analyzed under an inverted microscope (Caikon, XDS-900) using a 10× objective.

**Scratch migration assay**

4T1 cells were seeded in 24-well plates at 1 × 10⁵ cells/well. After cell attachment, a linear wound was created in each well using a sterile 200 μL pipette tip, followed by PBS washing to remove detached cells. Wound closure was photographed at 0 h and 48 h, and the wound area was quantified using NIH Image software.

**Figure legends:**

**Fig. S1** **The construction and in vitro evaluation of oncolytic adenovirus, rAd.DCN.CD40L**. **a** The schematic representation of oncolytic adenovirus encoding DCN or CD40L. **b-c** *Dcn* and *Cd40l* mRNA expression in TNBC cells was detected by RT-qPCR 24 h after infection (n=4). **d-e** Oncolytic adenoviral-induced cytotoxicity in TNBCs cells. The cells were infected with different viruses, and the cell survival was analyzed by MTS assay (n=6). The last column represents the combination treatment. The *P* values were determined by one-way ANOVA with Tukey’s post hoc test. The data are shown as the means ± SD of independent biological replicates. rAd, oncolytic adenovirus; PBS, phosphate-buffered saline; DCN, decorin; CD40L, CD40 ligand; TNBC, triple-negative breast cancer cells; RT-qPCR, real-time quantitative PCR.

**Fig. S2 Detection of intratumoral DCN and CD40L expression levels by ELISA. a** Tumor tissues was collected at the experimental endpoint, and intratumoral DCN levels were quantified by ELISA (n = 4). **b** Intratumoral CD40L levels were also measured by ELISA in endpoint tumor tissues (n = 4). The last column represents the combination treatment. The *P* values were determined by one-way ANOVA with Tukey’s post hoc test. The data are shown as the means ± SD of independent biological replicates.

**Fig. S3 Quantification of TGF-β- and EMT-related protein expression in tumor tissues.**

Quantitative analysis of western blot results showing the relative expression levels of TGF-β (**a**), β-catenin (**b**), E-cadherin (**c**), N-cadherin (**d**), vimentin (**e**), and Snail (**f**) in tumor tissues after different oncolytic adenovirus treatments (n = 4). Protein expression was normalized to β-actin and presented as fold change. The last column represents the combination treatment. The *P* values were determined by one-way ANOVA with Tukey’s post hoc test. The data are shown as the means ± SD of independent biological replicates.

**Fig. S4 Representative flow cytometry plots and quantification of CD8^+^T cells, and CD4^+^T cells. a** Representative flow cytometry plots of CD8⁺ and CD4⁺ T cells in the blood (n = 3). **b** Quantification of CD3⁺CD8⁺ T cells (n = 3). **c** Quantification of CD3⁺CD4⁺ T cells. The last column represents the combination treatment (n = 3). The *P* values were determined by one-way ANOVA with Tukey’s post hoc test. The data are shown as the means ± SD of independent biological replicates.
